# Topography shapes the structure, composition and function of tropical forest landscapes

**DOI:** 10.1111/ele.12964

**Published:** 2018-04-16

**Authors:** Tommaso Jucker, Boris Bongalov, David F. R. P. Burslem, Reuben Nilus, Michele Dalponte, Simon L. Lewis, Oliver L. Phillips, Lan Qie, David A. Coomes

**Affiliations:** ^1^ Department of Plant Sciences Forest Ecology and Conservation group University of Cambridge Cambridge UK; ^2^ CSIRO Land and Water 147 Underwood Avenue Floreat 6014 WA Australia; ^3^ School of Biological Sciences University of Aberdeen Cruickshank Building, St Machar Drive Aberdeen UK; ^4^ Sabah Forestry Department Forest Research Centre P.O. Box 1407 90715 Sandakan Sabah Malaysia; ^5^ Department of Sustainable Agro‐ecosystems and Bioresources, Research and Innovation Centre Fondazione E. Mach Via E. Mach 1 38010 San Michele all'Adige Italy; ^6^ School of Geography University of Leeds Leeds LS2 9JT UK; ^7^ Imperial College London, Silwood Park Campus Buckhusrt Road Ascot SL5 7PY UK

**Keywords:** Aboveground carbon density, airborne laser scanning (or LiDAR), biodiversity, canopy height, gap fraction, hyperspectral imaging, remote sensing, terrain elevation, slope and curvature, wood density

## Abstract

Topography is a key driver of tropical forest structure and composition, as it constrains local nutrient and hydraulic conditions within which trees grow. Yet, we do not fully understand how changes in forest physiognomy driven by topography impact other emergent properties of forests, such as their aboveground carbon density (ACD). Working in Borneo – at a site where 70‐m‐tall forests in alluvial valleys rapidly transition to stunted heath forests on nutrient‐depleted dip slopes – we combined field data with airborne laser scanning and hyperspectral imaging to characterise how topography shapes the vertical structure, wood density, diversity and ACD of nearly 15 km^2^ of old‐growth forest. We found that subtle differences in elevation – which control soil chemistry and hydrology – profoundly influenced the structure, composition and diversity of the canopy. Capturing these processes was critical to explaining landscape‐scale heterogeneity in ACD, highlighting how emerging remote sensing technologies can provide new insights into long‐standing ecological questions.

## Introduction

Tropical forests are among the most structurally complex, diverse and carbon‐rich ecosystems anywhere on earth (Pan *et al*. 2011; Klein *et al*. 2015; Ricklefs & He 2016). Yet, even within intact tropical landscapes, the structure, composition and function of forests can vary dramatically across very small spatial scales (Givnish 1999; John *et al*. 2007; Russo *et al*. 2008; Werner & Homeier 2015), often rivalling the degree of variation seen across broad environmental or biogeographic gradients (Quesada *et al*. 2012; Sullivan *et al*. 2017). Understanding what drives this fine‐scale heterogeneity in forest physiognomy is critical to forecasting how tropical forests will respond to global environmental change, as well as developing conservation and management strategies that maximise carbon‐biodiversity co‐benefits.

A key factor in maintaining heterogeneous forest landscapes is topography (Werner & Homeier 2015). Topographic features such as terrain relief, slope and curvature strongly influence local‐scale variation in soil chemistry, hydrology and microclimate (Tiessen *et al*. 1994; Chadwick & Asner 2016; Xia *et al*. 2016). As such, they directly constrain the conditions within which trees grow, driving environmental filtering, controlling species’ demographic rates (Baltzer *et al*. 2005; Russo *et al*. 2008; Anderse *et al*. 2014), and ultimately shaping the structure and composition of forest patches (Werner & Homeier 2015). For instance, on ridges and steep slopes, strong competition for nutrients and water favours species with life‐history traits geared towards maximising survival (Paoli 2006; Heineman *et al*. 2011; Holdaway *et al*. 2011). By contrast, forests in alluvial valleys are moulded by fierce competition for light, and generally develop taller, vertically stratified canopies (Paoli *et al*. 2008; Banin *et al*. 2012; Werner & Homeier 2015), while also maintaining higher productivity and turnover rates (Aiba *et al*. 2005; Stephenson *et al*. 2005; Quesada *et al*. 2012).

Yet, while concrete efforts have been made to link individual features of forest physiognomy to landscape‐scale variation in topography (Colgan *et al*. 2012; Detto *et al*. 2013; Swetnam *et al*. 2017), we continue to lack a complete picture of how structural and compositional attributes co‐vary along topographic gradients, and how this in turn shapes other emergent properties of forests such as their aboveground carbon density (ACD). A key challenge in this respect is that, by themselves, traditional ground‐based surveys are poorly suited to this task, particularly in the tropics where logistical constraints mean that plot networks are often unrepresentative of the wider landscape (Marvin *et al*. 2014). In this regard, airborne remote sensing promises to revolutionise the way we study forests by providing large‐scale coverage of key biophysical parameters at high resolution. For instance, airborne laser scanning (ALS, or LiDAR) can be used to simultaneously capture the 3D structure of both the canopy and the underlying terrain (Lefsky *et al*. 2002; Wulder *et al*. 2012; Detto *et al*. 2013), and is now routinely used as a tool to map ACD in tropical forests (Asner & Mascaro 2014; Réjou‐Méchain *et al*. 2015; Jucker *et al*. 2018). By coupling ALS with novel spectranomic approaches that are able to resolve the biochemical makeup of forest canopies based on how they reflect light (Townsend *et al*. 2008; Asner *et al*. 2015; Schneider *et al*. 2017), we are now in a position to comprehensively assess the relative importance of different topographic features in driving heterogeneity in the structure, composition and function of tropical forest landscapes.

**Figure 1:**
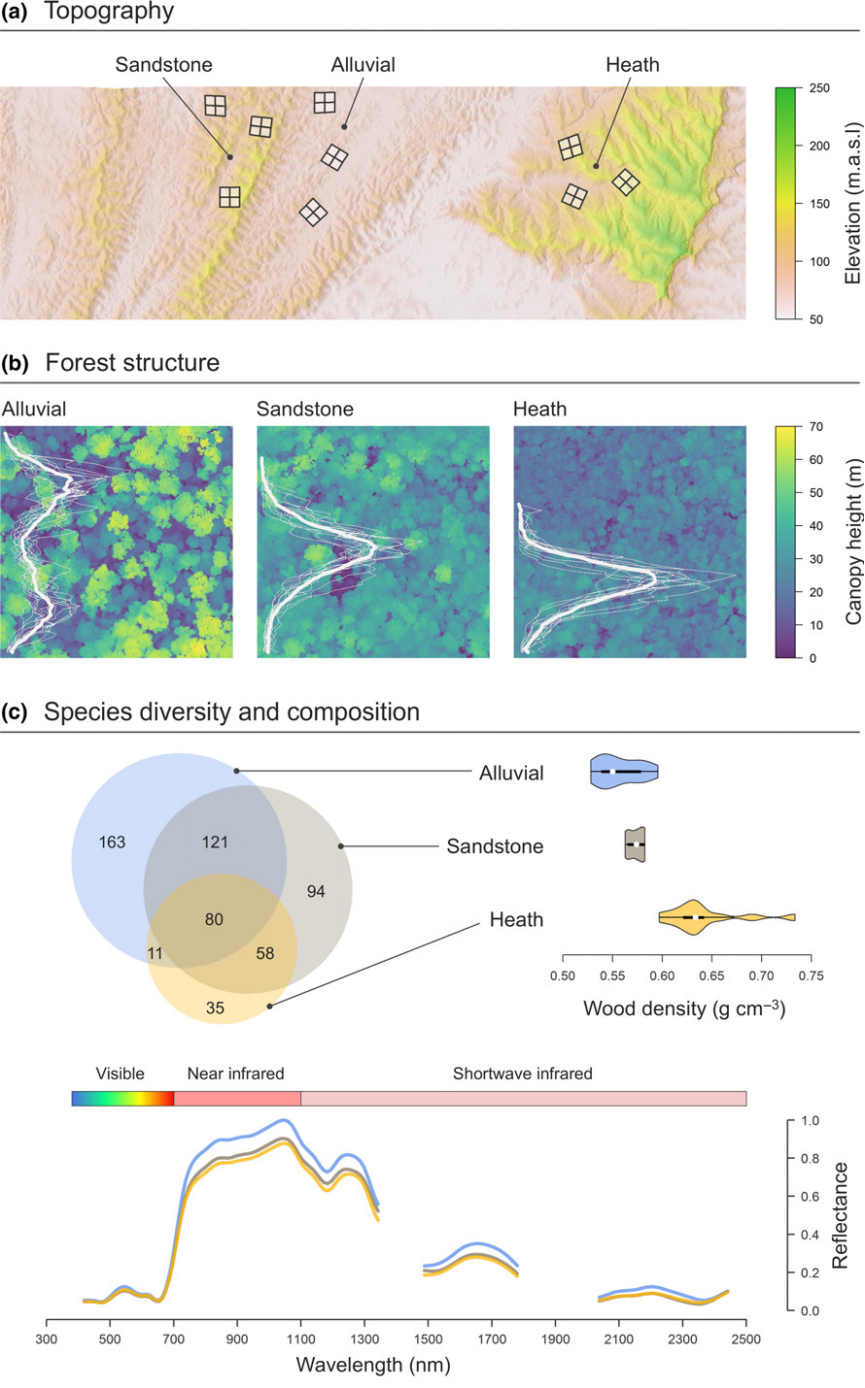
Variation in (a) topography, (b) forest structure and (c) species diversity and composition across Sepilok Forest Reserve. Panel (a) is a digital elevation model (DEM) of the study area obtained from the airborne laser scanning data (ALS). On it are superimposed the location 36 1‐ha permanent field plots (12 for each forest type). Panel (b) shows an example of the canopy height model (CHM) for each of the three forest types. The superimposed white curves correspond to the vertical ALS return profiles of the contrasting forest types. In (c), the Venn diagram illustrates how species richness and composition vary among the three forest types, while the violin plots captures differences in community‐weighted mean wood density among forest types. The reflectance profiles of the three forest types obtained from the airborne hyperspectral imagery are shown below.

Working at Sepilok Forest Reserve in Malaysian Borneo, we combine field data from 36 1‐ha plots with ALS and hyperspectral imagery to determine whether topography explains the astonishing degree of variation in forest structure, composition and ACD found at this site (Fig. 1). To start, we develop statistical models calibrated against field data to estimate tree species richness, wood density and ACD at 1‐ha resolution across approximately 15 km^2^ of old‐growth forest using remotely sensed attributes as inputs. We then contrast two alternative approaches for unpacking how topography shapes landscape‐scale heterogeneity in ACD. The first involves fitting simple regression models that relate variation in ACD directly to terrain elevation, slope and curvature – mimicking previous attempts to characterise ACD–topography relationships (e.g. Colgan *et al*. 2012; Swetnam *et al*. 2017). The second uses structural equation models (SEM) to determine how variation in ACD along topographic gradients is mediated by changes in the vertical structure, functional composition and diversity of forest patches. By doing so we shed light on the underlying processes that shape what are often complex associations between ACD and topography in tropical forests.

## Materials and Methods

### Study site

Sepilok Forest Reserve (5°10’ N 117°56’ E) is a remnant of lowland tropical rainforest on the east coast of the Malaysian state of Sabah in Borneo. The reserve was founded by the Sabah Forest Department in 1931 – making it one of the oldest protected areas in Southeast Asia – and covers approximately 4500 ha of forest ranging in elevation between 50 and 250 m.a.s.l. (Fox 1973). Much of Sepilok has never been commercially exploited, although areas to the northeast and south of the reserve were selectively logged until 1957 (Dent *et al*. 2006). Three distinct forest types are found within the reserve: alluvial dipterocarp forest in the valleys; sandstone dipterocarp forests on dissected hillsides and crests; and heath forests that dominate podzols associated with the dip slopes of cuesta. Previous research conducted at Sepilok has highlighted how these distinct forest types differ not only in their species composition and diversity, but also in terms of nutrient cycling, forest structure and ACD (Fig. 1; Austin & Greig‐Smith 1968; Dent *et al*. 2006; Coomes *et al*. 2017).

### Permanent plot data

Nine permanent 4‐ha forest plots were established within the Sepilok reserve in 2000–01, three for each forest type (Fig. 1). These were re‐censused in 2013–15, at which point all stems with a diameter (D; cm) ≥ 5 cm were measured and identified to species (or closest known taxonomic unit; *n* = 45 214 stems). For a subset of trees in each plot, height and crown size were also measured using a laser range finder. For the purposes of this analysis, each 4‐ha plot was subdivided into 1‐ha subplots (*n* = 36). The corners of the 1‐ha plots were geolocated using a differential GPS (Geneq SXBlue II). Note that despite subplots being adjacent to each other, we found no evidence of significant residual spatial autocorrelation for any of the models presented hereafter (tested using Moran's I statistic).

For each 1‐ha plot, we used the field data to calculate three plot‐level attributes: ACD (Mg C ha^−1^), the community‐weighted mean wood density (WD; g cm^−3^) and tree species richness. ACD was estimated by first calculating the aboveground biomass (AGB; kg) of individual trees using Chave *et al*.'s (2014) pantropical biomass equation – where $AGB=0.067\times {\left(D^{2}\times H\times WD\right)}^{0.976}$ – and then summing the AGB of all trees within a plot after applying a carbon content conversion factor of 0.47 (see Jucker *et al*. (2018) for details). Plot‐level WD was quantified as $\sum^{B}A_{\mathrm{ij}}\times WD_{i}$, where BA_ij_ is the relative basal area of species *i* in plot *j*, and WD_i_ is the mean wood density of species *i* which we obtained from the global wood density database (Chave *et al*. 2009). For stems not identified to species (9%) or those belonging to species not represented in the database (41%), genus‐ or plot‐level mean WD values were used instead (43 and 7% of stems respectively).

Prior to estimating species richness, we used information on the relative position and crown size of trees within the 1‐ha plots to classify stems as either emergent or belonging to the understorey (see Appendix S1). Because hyperspectral imagery only captures the surface of the canopy, we then calculated the species richness of emergent trees in each plot as a measure of canopy diversity. We reason that focusing on emergent trees provides a better representation of the canopy as seen from above, but note that estimates of tree species richness calculated in this way were strongly related to ones obtained using data for all stems (Pearson's correlation = 0.91).

### Airborne laser scanning data

ALS data were acquired in November 2014 using a Leica ALS50‐II LiDAR sensor flown by NERC's Airborne Research Facility. Data acquisition parameters and processing are described in detail in Coomes *et al*. (2017). Briefly, the data were obtained as a discretised point cloud, with up to four returns recorded per pulse and an average density of 18.5 pulses m^−2^. Points were classified into ground and non‐ground returns using the LAStools software (http://rapidlasso.com/lastools), and a digital elevation model (DEM) was fit to the ground returns to produce a 1‐m resolution raster. The DEM was then subtracted from the elevations of all non‐ground returns to produce a normalised point cloud, from which a 1‐m resolution canopy height model (CHM) was constructed by averaging the first returns. All further processing of the ALS data was done using the *raster* package in R (Hijmans 2016; R Core Development Team 2016).

We used the ALS data to characterise the canopy structure and topography of both the study plots and of the wider Sepilok landscape, which we sampled using a regular grid comprising of 1‐ha cells covering the extent of the flight path (*n* = 1853 cells). From the CHM, we extracted two canopy structural metrics which previous work has shown to be key determinants of ACD at Sepilok and across Sabah more widely (Coomes *et al*. 2017; Jucker *et al*. 2018): mean top‐of‐canopy height (TCH; m) and gap fraction at 20 m aboveground. TCH is the mean height of the pixels which make up the surface of the CHM, while gap fraction is the proportional area with no crown cover at a specific height aboveground (20 m in this case), and captures heterogeneity in canopy height and density.

From the DEM, we calculated three topographic variables which are known to influence soil structure, chemistry and hydrology (Detto *et al*. 2013; Chadwick & Asner 2016): elevation within the landscape (m.a.s.l.), terrain slope (degrees) and topographic position index (TPI), which describes the curvature of the terrain relative to the surrounding landscape and ranges from negative where the terrain is concave (i.e. gulleys) to positive where it is convex (i.e. ridges). Elevation was calculated by taking the mean value of the DEM within each 1‐ha grid cell. For slope and TPI a two‐step approach was used instead. First, we coarsened the resolution of the DEM to 10 m via spatial averaging. Slope and TPI were then calculated at this scale, following which a mean value for each 1‐ha grid cell was taken. This ensured that slope and TPI estimates did not exhibit extreme localised values as often occurs when using a very high resolution terrain model as input.

### Hyperspectral imagery

Hyperspectral imagery was obtained with an AisaFENIX sensor mounted alongside the laser scanner. The sensor measures light reflectance between 380 and 2500 nm, collecting data across 622 spectral bands at 1‐m spatial resolution. To improve the signal‐to‐noise ratio of the data, we spectrally resampled the images by averaging every three bands in the visible and near infrared regions, and two bands in the shortwave infrared (resulting in a total of 189 spectral bands). All further processing followed the guidelines outlined in Asner *et al*. (2015). First, we applied atmospheric and BRDF correction using the MODTRAN radiative transfer model as implemented in the ‘rugged terrain’ module of ATCOR‐4. We then removed bands < 410 nm and those > 2450 nm, as they are known to be noisy, as well as water absorption bands between 1350 and 1480 nm and 1780–2032 nm, leaving a total of 147 spectral bands. Lastly, poorly illuminated pixels (identified by applying a ray‐tracing algorithm to the ALS data) and those with a Normalized Difference Vegetation Index (NDVI) < 0.8 were masked out.

### Upscaling estimates of species richness and wood density from plot to landscape scale

To upscale estimates of species richness and WD across the Sepilok landscape, we used partial least squares regression (PLSR) models to relate plot‐level values derived from the field data to remotely sensed spectral attributes (Asner *et al*. 2015). The fitted PLSR models were then used to predict both species richness and WD across the landscape at 1‐ha resolution using the regular sampling grid described above.

#### Tree species richness

Previous attempts to estimate tree diversity from spectral data have largely focused on the idea that the variability in spectral signatures recorded in a given area should reflect the diversity of species found within it (Rocchini *et al*. 2004, 2010; Carlson *et al*. 2007; Laurin *et al*. 2014). Building on this work, for each 1‐ha plot, we used the hyperspectral imagery to calculate the standard deviation of the reflectance of each spectral band (*n* = 147) across all 1‐m pixels found within the extent of the plot. These standard deviation values were then used as predictors of plot‐level tree species richness in the PLSR models.

We chose PLSR as a modelling approach as it is ideally suited to dealing with the high dimensionality and collinearity typical of hyperspectral data, which PLSR reduces to a small number of components (or latent variables) to be used as predictors (Mevik & Wehrens 2007). PLSR models were fit using the *pls* package in R (Mevik & Wehrens 2007). We used the root mean squared error (RMSE) of prediction calculated on the basis of leave‐one‐out cross‐validation to determine the optimal number of components in the model (Asner *et al*. 2015; see Appendix S2 for details). The predictive accuracy of the models was assessed using the coefficient of determination (*R* ^2^) calculated on the validation data. For model fitting, we used data from 33 of the 36 1‐ha plots, as following the data processing and quality control steps applied to the hyperspectral imagery which are described above, three plots had insufficient coverage to reliably estimate the spectral indices used in the models (i.e. < 1000 viable 1‐m pixels per plot from which to calculate the standard deviation values for each spectral band). The same cut‐off was applied when using the fitted models to estimate species richness across the landscape, resulting in a total of 1369 1‐ha grid cells with sufficiently high‐quality spectral data for all further analyses.

#### Wood density

Accurately estimating plot‐level WD from remotely sensed data has proven challenging, and remains a major source of uncertainty in efforts to map forest carbon stocks (Mitchard *et al*. 2014). Based on the observation that community‐mean WD tends to increase during forest succession, previous work has attempted to estimate WD from ALS‐derived metrics such as TCH, but with mixed results (Asner & Mascaro 2014; Jucker *et al*. 2018). Here, we take a different approach which assumes that because leaf and stem traits are generally coordinated in trees (Méndez‐Alonzo *et al*. 2012; Nolf *et al*. 2015; Zeballos *et al*. 2017; although see Baraloto *et al*. 2010), we can use hyperspectral imagery – which has been shown to reliably capture leaf properties (e.g. Asner *et al*. 2015) – to also estimate WD. Specifically, for each 1‐ha plot, we used the hyperspectral imagery to calculate the mean reflectance value of each spectral band (*n* = 147) across all 1‐m pixels found within the extent of the plot. These mean reflectance values were then used as predictors of plot‐level WD in the PLSR models. Model fitting, validation and upscaling across the landscape followed the same approach described above for species richness.

### Upscaling estimates of ACD from plot to landscape scale

We used the equation developed in Coomes *et al*. (2017) for estimating ACD at Sepilok from ALS data as a starting point for upscaling carbon stocks across the landscape. This work builds on Asner & Mascaro (2014), where the authors develop a two‐step approach for calibrating a general model relating ACD to ALS data. First, plot‐level estimates of ACD, basal area (BA; m^2^ ha^−1^) and WD are combined with ALS‐derived measurements of TCH to parameterize the following power‐law function: $ACD=\rho_{0}\times BA^{\rho_{1}}\times TCH^{\rho_{2}}\times WD^{\rho_{3}}$, where ρ_*0–3*_ are parameters to be estimated from the data. Then, two additional sub‐models are fit for estimating BA and WD from ALS‐derived predictors. By substituting these subroutines into the main equation, ACD can then be estimated directly from ALS data.

In Asner & Mascaro (2014) both BA and WD were estimated from TCH. However, Coomes *et al*. (2017) found gap fraction at 20 m aboveground to be a much stronger predictor of BA at Sepilok, the two being negatively correlated with one another. Following this approach, we modelled BA as a power‐law function of gap fraction. Additionally, we also replaced the sub‐model relating WD to TCH – which previous work has shown to perform poorly for forests in Sabah (Jucker *et al*. 2018) – with estimates of WD derived directly from hyperspectral imagery as described above. We evaluated the accuracy of ACD estimates obtained in this way by calculating the RMSE and average systematic error (or bias) of the predictions using a leave‐one‐out cross‐validation procedure, and used the model to estimate ACD for all 1369 1‐ha grid cells covering the Sepilok landscape for which both ALS and high‐quality hyperspectral imagery were available. Parameter estimates for both the ACD and BA equations developed for Sepilok are provided in Appendix S3.

### Landscape‐scale drivers of forest structure, wood density, species richness and ACD

To characterise how topography influences variation in ACD across Sepilok, we started by fitting a multiple regression model relating ACD to elevation, slope and TPI using data from the 1369 1‐ha grid cells distributed across the reserve. The model also included species richness as a predictor, to test whether ACD and diversity co‐vary positively across the landscape (Poorter *et al*. 2015; Sullivan *et al*. 2017). A preliminary inspection of the data highlighted clear differences between forests on level ground (TPI ≈ 0) and ones on either ridges or gullies (TPI ≠ 0), and suggested that ACD might vary nonlinearly with elevation, slope and species richness. We therefore modelled the effects of terrain curvature on ACD using absolute values of TPI, and included quadratic terms for elevation, slope and species richness in the regression. Principal components analysis was used to visualise the combined effects of topography and species richness on ACD.

We then contrasted this simple multiple regression approach with a more nuanced one that uses SEM to tease apart how topography influences the structure, traits composition and diversity of forests at Sepilok, and how this in turn shapes heterogeneity in ACD across the landscape. In these models, ACD was expressed as a composite variable of TCH, gap fraction and WD (which together were used to estimate ACD), allowing us to characterise how topographic effects on ACD are mediated though changes in canopy structure and composition (Grace *et al*. 2010). To identify these indirect effects, the SEM included direct pathways linking topographic features (elevation, terrain slope and TPI) to both structural (TCH and gap fraction) and compositional attributes (WD and species richness). Our expectation was that areas at higher elevation within the landscape, on steep slopes and/or on ridges would exhibit shorter and more compact canopies, higher WD and lower tree species richness compared to low‐lying valleys associated with alluvial soils – thus resulting in potentially complex, nonlinear associations between ACD and topography. The models also included covariance terms linking structural and compositional attributes of the canopy, as we expect topography to influence these in coordinated ways (e.g. we predict that areas characterised by forests with high community‐mean WD would have lower TCH values than ones where resource‐acquisitive species with lower WD are more prevalent, as these typically invest heavily in height growth to escape shaded understoreys).

SEM were fit using the *lavaan* R package (Rosseel 2012), which allows latent and composite variables (e.g. ACD in our case) to be incorporated in the model. ACD, TCH, gap fraction and WD were log‐transformed to replicate the structure of the plot‐level models described above, as was species richness to account for the bounded nature of the data. Following the multiple regression modelling of ACD, we took the absolute values of TPI as a model predictor. Prior to model fitting, all variables were standardised by calculating *z*‐scores to aid model convergence (Rosseel 2012). Standardised path coefficients (and associated *P*‐values) were calculated for individual pathways in the model to assess their relative contribution to the explained variance. Model selection was performed on the basis of AIC, with step‐wise removal of non‐significant pathways (*P* > 0.05). Model fit was assessed using a Chi‐squared test and associated *P*‐value (where *P* > 0.05 indicates good agreement between the model and the data; Grace *et al*. 2010). In addition to fitting a single SEM to all gridded data together, we also explored whether associations between topography, canopy structure and composition were broadly consistent within the three forest types present at Sepilok. To do this, we used an existing map detailing the spatial distribution of forest types within the reserve (see Appendix S4) to assign a forest type class to each 1‐ha grid cell. We then fit separate SEM to data from each forest type following the same model structure described above.

## Results

### Estimating species richness, wood density and ACD from ALS and hyperspectral data

**Figure 2:**
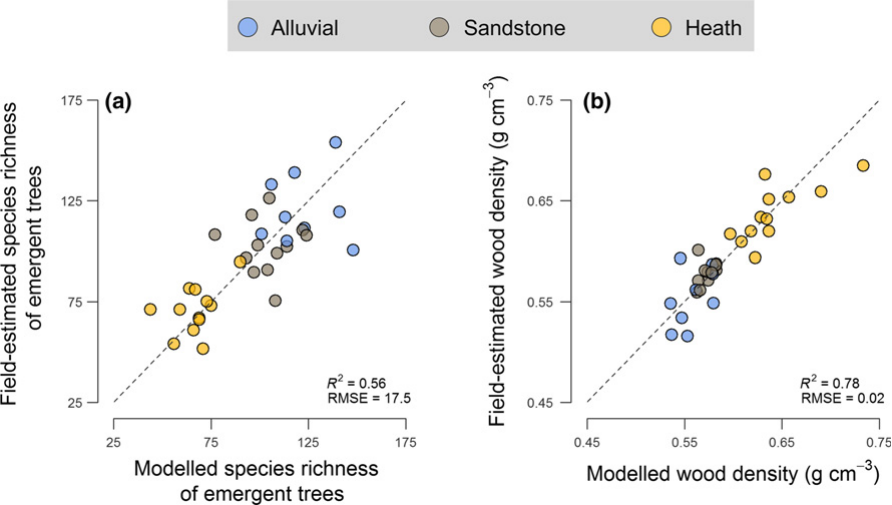
Predictive accuracy of partial least squares regression (PLSR) models of (a) species richness of emergent trees and (b) community‐weighted mean wood density. Predicted values from the PLSR models for each of the 1‐ha permanent plots (*n* = 33) are based on leave‐one‐out cross‐validation. Points are grouped according to forest type based on their colour. Cross‐validated *R* ^2^ and RMSE values for both models are reported in the bottom right‐hand corner of each panel.

PLSR models fit to plot and hyperspectral data provided reasonably accurate and unbiased estimates of species richness (Fig. 2a), with an *R* ^2^ of 0.56 based on leave‐one‐out cross‐validation (RMSE = 17.5, or 13.5% of the mean). PLSR performed even better for WD (Fig. 2b), explaining 78% of the variation in the validation data (RMSE = 0.021 g cm^−3^, or 3.5% of the mean). For ACD, modifying the regression modelling approach developed by Coomes *et al*. (2017) to include a subroutine for estimating WD from hyperspectral imagery yielded accurate and unbiased estimates of ACD (RMSE = 27.7 Mg C ha^−1^, or 12.7% of the mean, bias = 0.7%; compared to an RMSE of 29.0 Mg C ha^−1^ reported for the original model). Applying these three models across Sepilok highlighted considerable variation in emergent tree species richness (mean = 108 species ha^−1^; range = 40–197 species ha^−1^), community‐mean WD (mean = 0.57 g cm^−3^; range = 0.49–0.74 g cm^−3^) and ACD (mean = 224.6 Mg C ha^−1^; range = 126.1–361.2 Mg C ha^−1^) both across the landscape and within forest types.

### Topographic drivers of forest structure, wood density, species richness and ACD

**Figure 3:**
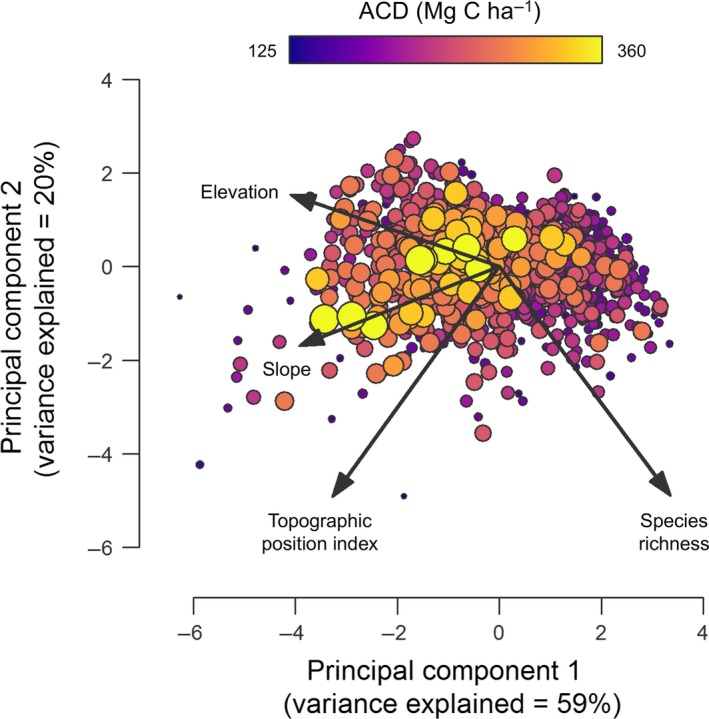
Principal components analysis (PCA) illustrating how elevation, slope, topographic position index and species richness co‐vary with aboveground carbon density (ACD) across the 1‐ha grid cells that span the Sepilok landscape (*n* = 1369). The first two principal components of the PCA (which together explained 79% of the variation in elevation, slope, TPI and species richness) are plotted, with the size and colour of the points reflecting the ACD of that particular 1‐ha grid cell. Grid cells with high ACD values were strongly clustered towards the centre of the ordination, reflecting the fact that ACD peaked in areas at mid elevations, on moderate but not extreme slopes, and at intermediate levels of tree species richness.

Multiple regression modelling revealed strong, yet complex associations between ACD, topography and tree species richness across the Sepilok landscape (Fig. 3). The best fitting model explained 23% of the variation in ACD, and included quadratic terms for elevation, slope and species richness (all of which had negative coefficients in the model, indicating that ACD tended to peak at mid elevations within the landscape, on relatively steep, but not extreme slopes and at intermediate levels of tree diversity; Fig. 3). The model also captured a negative relationship between ACD and absolute TPI values, consistent with ACD being highest on relatively flat terrain where TPI ≈ 0 (see Appendix S5 for a summary of all model coefficients). Of the three topographic attributes, ACD was most strongly associated with elevation and reached a peak at *c*. 136 m.a.s.l. before declining again.

**Figure 4:**
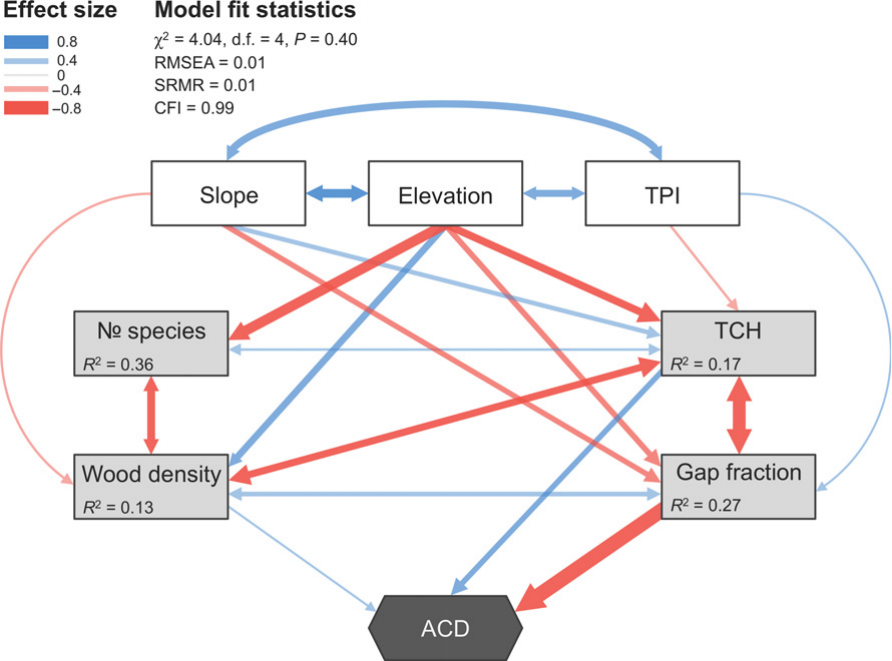
Structural equation model relating variation in tree species richness (№ species), wood density, top‐of‐canopy height (TCH) and gap fraction at 20 m aboveground to terrain slope, elevation and topographic position index (TPI). Aboveground carbon density (ACD) was modelled as a composite variable of TCH, gap fraction and wood density. Exogenous variables are represented by white boxes, while endogenous variables are shaded in grey. *R* ^2^ values are reported for each endogenous variable. The width of the arrows reflects the strength of the pathway and is proportional to the standardised path coefficient as shown in the legend. Blue arrows denote positive relationships, while red arrows correspond to negative ones. Covariance terms in the model are represented by bi‐directional arrows. All reported pathways were significant to *P *≤ 0.001. Summary model fit statistics, including the root mean square error of approximation (RMSEA; target value < 0.05), the standardised root mean square residual (SRMR; target value < 0.10) and the comparative fit index (CFI; target value > 0.90), are reported in the top left‐hand corner.

**Figure 5:**
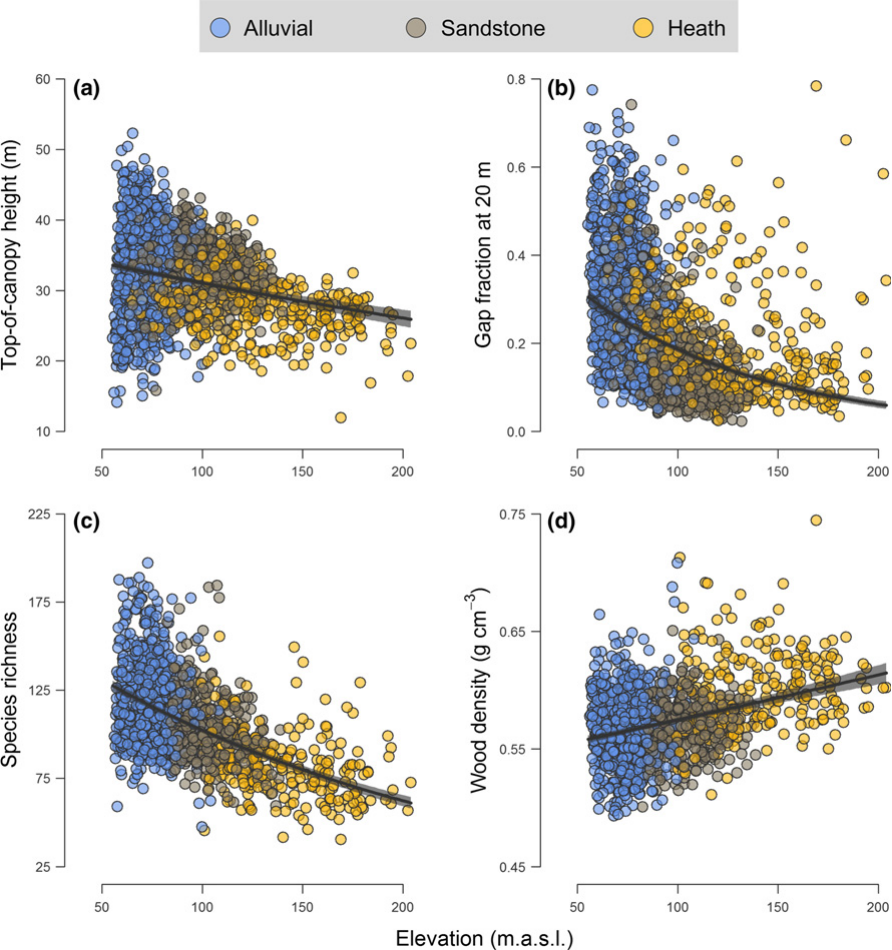
Variation in (a) top‐of‐canopy height, (b) gap fraction, (c) species richness and (d) community‐weighted mean wood density in relation to elevation across the Sepilok Forest Reserve. Each point corresponds to a 1‐ha grid cell (*n* = 1369), with fitted curves (± 99% confidence intervals) highlighting bivariate relationships captured by the structural equation model depicted in Fig. 4. Points are grouped according to forest type based on their colour.

**Figure 6:**
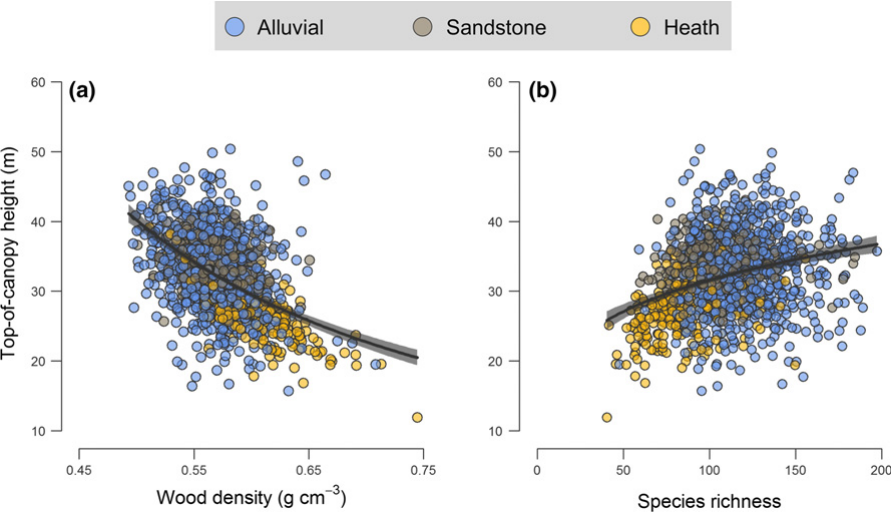
Variation in top‐of‐canopy height as a function of (a) community‐weighted mean wood density and (b) species richness across the Sepilok Forest Reserve. Each point corresponds to a 1‐ha grid cell (*n *= 1369), with fitted curves (± 99% confidence intervals) highlighting bivariate relationships captured by the structural equation model depicted in Fig. 4. Points are grouped according to forest type based on their colour.

SEM provided key insights into the underlying processes that promote landscape‐scale heterogeneity in ACD, as they revealed a clear influence of topography in driving variation in the structure, functional composition and diversity of forests at Sepilok both across and within forest types (Fig. 4). Overall, the SEM parameterized using data from all 1‐ha grid cells together fit the data well (χ^2^ = 4.04; *P* = 0.40), with topographic variables alone explaining 36% of the variation in tree species richness, 13% of that in WD, 17% for TCH and 27% for gap fraction at 20 m aboveground (Fig. 4). While all three topographic attributes contributed significantly to shaping forest structure, diversity and trait composition at Sepilok, elevation within the landscape emerged as the strongest of the three drivers (Figs 4 and 5). As predicted, both TCH and species richness declined progressively with elevation (Fig. 5a and c), while WD showed the opposite pattern and peaked at higher elevations within the landscape (Fig. 5d). Yet, despite the fact that alluvial valleys had the tallest canopies, they also tended to have higher gap fractions at 20 m aboveground compared to forests at mid elevations and above (Fig. 5b). This helps explain why ACD was not highest in tall, low‐lying alluvial forests, as gap fraction was by far the strongest predictor of ACD at Sepilok (Fig. 4). Lastly, the SEM also revealed strong links between compositional and structural elements of forests at Sepilok that help contextualise why ACD varies the way it does across the landscape (Fig. 6). In particular, we found that forest patches with shorter canopies were generally dominated by species with high WD (Fig. 6a) – further dampening the relationship between TCH and ACD – whereas taller forests not only had lower mean WD but were also generally more diverse (Fig. 6b). These patterns linking variation in forest structure and composition to topography were remarkably consistent even when data were analysed separately for each forest type (see Appendix S6).

## Discussion

Topography emerged as a strong driver of heterogeneity in ACD across the Sepilok landscape, matching what previous studies have reported for a range of forest types (Colgan *et al*. 2012; Werner & Homeier 2015; Swetnam *et al*. 2017). However, our analysis also revealed that associations between ACD and terrain features were highly complex and nonlinear (Fig. 3), making them impossible to interpret when analysed in isolation. Instead, understanding the processes that underpin these patterns required a more nuanced approach that accounts for the combined effects of topography on the structure, trait composition and diversity of forest patches, and how these in turn interact to determine ACD (Fig. 4).

### Topographic controls over forest structure, composition and function

Canopy vertical structure and composition varied dramatically with topography at Sepilok both within and across forest types (Fig. 4), reflecting strong shifts in species composition and demographic profiles of communities that unfold along topo‐edaphic gradients (Fig. 1; Baltzer *et al*. 2005; Russo *et al*. 2008; Anderse *et al*. 2014). These shifts in forest structure and composition occurred along a continuum rather than at sharply defined transition zones between otherwise homogeneous forest types (Appendix S6). TCH declined progressively with elevation within the landscape (Fig. 5a), with maximum attained TCH values decreasing by 1.5 m for every 10 m of elevation gain. This pattern is consistent with empirical evidence highlighting strong shifts in carbon allocation strategies and crown architecture of trees as soil nutrients and water availability become increasingly limiting, as typically occurs on ridges, steep slopes and at higher elevations within the landscape (Werner & Homeier 2015). Under these conditions, forests become dominated by species with conservative life‐history strategies geared towards survival rather than rapid height growth (e.g. high root:shoot ratios, high WD, physical/chemical defences against herbivores; King *et al*. 2006; Baltzer & Thomas 2010; Heineman *et al*. 2011; Cosme *et al*. 2017; Greenwood *et al*. 2017). These trade‐offs were very apparent at Sepilok, where canopy height and mean WD were strongly negatively correlated across the landscape (Fig. 6a).

Despite TCH peaking in alluvial valleys, we also found that low‐lying forests tended to have higher gap fractions at 20 m aboveground compared to ones at mid elevations and above, where canopy structure became more uniform and compact (Fig. 5b). This pattern further highlights how subtle differences in elevation can contribute to driving strong changes in the composition and dynamics of communities within the landscape (Werner & Homeier 2015), which ultimately underpin the variation in canopy structure seen in the ALS data (Fig. 1b). Low‐lying, alluvial valleys in Bornean forests are home to a wide variety of tree species (Fig. 1c), including fast‐growing dipterocarps that invest heavily in height growth to escape shaded understories and can become exceptionally tall (exceeding 90 m in rare cases; Ghazoul 2016). However, in addition to being highly productive (Banin *et al*. 2014), these forests also exhibit high turnover rates (Stephenson *et al*. 2005; Russo *et al*. 2008). In particular, large, emergent dipterocarps – which are susceptible to strong winds and extreme flooding events, due to their large crowns, shallow root systems and low WD (Proctor *et al*. 2001; King *et al*. 2006; Margrove *et al*. 2015) – can create large canopy gaps when they die. The net result of these demographic processes is that when nutrients are not limiting to growth, forests tend to develop taller, but also more structurally complex canopies.

When the effects of topography on forest structure and composition were scaled to ACD, we found that the tallest forests were not necessarily the most carbon dense. Instead, ACD was primarily associated with low gap fraction, and tended to peak at mid elevations within the landscape in densely packed sandstone hill forests whose canopies are 4.7 m shorter on average than those in alluvial valleys (Fig. 1b). Further dampening the relationship between ACD and TCH is the fact that WD was negatively correlated with TCH (Fig. 6a), and tended to increase progressively with elevation (Fig. 5d). Consequently, while the TCH of heath forests was less than ⅔ that of alluvial ones (Fig. 1b), the difference in ACD between the two forest types was only 13% (despite them having almost identical basal areas). Similar patterns have been found elsewhere along large‐scale edaphic gradients (e.g. east‐to‐west soil fertility gradients in the Amazon; Quesada *et al*. 2012), but our study highlights how the same processes can play out within a few hundred metres. Nevertheless, we note that while topographic features are clearly important in driving variation in ACD within forest landscapes, the strength, shape and complexity of these relationships is likely to be – at least in part – site‐specific (McEwan *et al*. 2011; Detto *et al*. 2013; Réjou‐Méchain *et al*. 2015), reflecting complex and context‐dependent associations between topography and edaphic processes.

The lack of a strong relationship between ACD and TCH contrasts with what is typically found along successional trajectories, where both ACD and WD tend to increase as canopies grow taller with age (Asner & Mascaro 2014). However, how much ACD can be accumulated by a given patch of forest ultimately depends on the net outcome of demographic processes linked to tree growth, recruitment and mortality. At Sepilok, the optimal balance is struck at mid elevations and on moderate‐to‐steep slopes that characterise sandstone hill forests, where low turnover rates allow for tightly packed and carbon‐rich forests to form (Nilus 2004). This underscores why TCH alone cannot always be used to reliably predict ACD in mature forests (Duncanson *et al*. 2015; Coomes *et al*. 2017), highlighting the need for new general models for estimating ACD from ALS data (Jucker *et al*. 2018). An integral part of this process will involve refining current approaches to estimating WD from remotely sensed data. Our results highlighting a strong link between canopy reflectance profiles and community‐mean WD values are promising in this respect (Fig. 2b). Yet, whether similar patterns also hold for other forest types – and if so, the extent to which these relationships can be traced back to coordination between species’ leaf and stem traits – remains to be determined.

### Unpacking carbon–diversity relationships in tropical forests

In addition to shaping canopy structure and composition, topography also influenced patterns of tree diversity across Sepilok (Fig. 4). Nutrient‐rich alluvial valleys supported the most diverse forests (Fig. 1c), likely reflecting the large range of shade‐tolerance niches created when combining extremely tall canopies with fast turnover rates (Coomes *et al*. 2009; Dent & Burslem 2016). Moving uphill resulted in a steady decline in tree diversity (Fig. 5c), which is consistent with some (Givnish 1999; Aiba *et al*. 2005; Laurance *et al*. 2010) but not all studies that have explored how diversity varies along soil fertility gradients in tropical forests (e.g. Huston 1980). These discrepancies likely depend on the degree to which soil fertility and carbon dynamics are coupled within a site: when nutrient‐rich substrates are associated with fast turnover rates – as is the case at Sepilok – soil fertility is expected to promote tree diversity. When this association is weaker, nutrient‐rich soils can become dominated by relatively few, highly competitive species.

When relating ACD to tree diversity across Sepilok, we found that ACD tended to peak at intermediate levels of tree species richness before declining again in the most diverse forest patches (Fig. 3). While this seemingly contrasts with recent syntheses showing that diverse forests are generally more productive than species‐poor ones (Liang *et al*. 2016), it closely matches the results of two pantropical analyses that explored carbon–diversity relationships using data from 1‐ha field plots (Chisholm *et al*. 2013; Sullivan *et al*. 2017). Both studies found that the ecological processes that promote positive ACD–diversity relationships are most relevant at extremely small, neighbourhood scales (0.04 ha and below). Instead, at scales approaching 1‐ha and above, variation in both ACD and tree diversity is largely driven by changes in species composition that unfold along environmental and successional gradients. This makes it incredibly challenging to predict under what circumstances we should expect carbon–diversity associations to be positive at spatial scales that are relevant for management and conservation. In this respect, emerging remote sensing technologies provide a solution to bypassing this issue entirely by allowing us to simultaneously capture how both diversity and ACD vary across entire landscapes.

## Author contributions

DAC coordinated the NERC airborne surveys of Sabah. TJ and DAC designed the study; TJ and BB processed the airborne imagery with assistance from MD, while all other authors contributed field data; TJ analysed the data and wrote the first draft of the manuscript, with all other authors contributing to revisions.

## Data Availability

Data used in this study are available at: https://doi.org/10.6084/m9.figshare.5998616.v1
