# Supplementary material for: Topography shapes the structure, composition and function of tropical forest landscapes
Source: Ecol Lett. 2018 Apr 16;21(7):989–1000. doi: 10.1111/ele.12964 (PMC6849614; doi:10.1111/ele.12964)
Supplement: Supplementary file 1 [file ELE-21-989-s001.docx]

**Supporting information**

[Appendix S1 | Quantifying species richness of emergent trees 2](#_Toc509212793)

[Appendix S2 | Selecting number of components for partial least squares regression models 4](#_Toc509212794)

[Appendix S3 | Estimating ACD and basal area from ALS-derived metrics 5](#_Toc509212795)

[Appendix S4 | Landscape-scale maps of topographic, structural and compositional variables 6](#_Toc509212796)

[Appendix S5 | Summary output of best-fitting multiple regression model of ACD 7](#_Toc509212797)

[Appendix S6 | Forest type-specific structural equation models 8](#_Toc509212798)

[References 9](#_Toc509212799)

## Appendix S1 | Quantifying species richness of emergent trees

Differently from airborne laser scanning which is able to penetrate the canopy and provide information on the vertical distribution of leaves, branches and stems, hyperspectral imagery only captures light reflectance from objects that are in direct line-of-sight of the sensor. Consequently, when attempting to estimate tree species richness from hyperspectral imagery, we are confronted with the issue that a portion of trees are not visible to the sensor (particularly in structurally complex and multi-layered tropical forests). To address this mismatch between what we see from the air and what is measured on the ground, we used information on the relative position and size of trees within the 36 1 ha plots to first categorize each tree as either emergent or overtopped, and then used these data to estimate the species richness of emergent trees only. Below we describe the process by which emergent tree species richness was estimated for each plot and explore how closely this metric relates to species richness calculated using data for all tress.

Because trees crowns will often overlap vertically (and to a lesser degree horizontally), the cumulative crown area of all trees within a given plot will often exceed the ground area of the plot itself. Yet only a foliated area equal to the ground area of the plot is visible to the hyperspectral sensor. Following this reasoning, we used information on the size and position of each stem to identify those trees whose crowns form the emergent canopy within each plot and are therefore in line-of-sight of the sensor. Specifically, within each of the 36 1 ha plots, all stems with a diameter at breast height (*D*, in cm) greater than 5 cm were measured, classified to species (or closest taxonomic unit), and assigned to a 10 × 10 m subplot (45,214 stems in total). Using these data, we first estimated the crown area (*CA*, in m^2^) of each tree as a function of its stem diameter using a locally-calibrated allometric equation developed in Coomes *et al.* (2017):

| $CA=0.182\times D^{1.531}$ |  | (eqn S1) |
| --- | --- | --- |

Within each 10 × 10 m subplot we then ranked trees according to size (from largest to smallest, in terms of *CA*) and then progressively summed their *CA* until it equalled (or first exceeded) the ground area of the subplot (i.e., 100 m^2^). The collection of largest trees within each subplot with cumulative *CA* ≤ 100 m^2^ were classified as emergent, while all other trees within the subplot were assigned to the understorey. In cases when the *CA* of the single largest tree in the subplot was ≥ 100 m^2^, only that tree was classified as emergent. Note that because tree locations were only known to within a 10 × 10 m subplot, and because tree crowns can overlap between adjacent subplots, in some cases our routine may misclassify trees as emergent or understorey. That being said, when averaged across all 10 × 10 m subplot within a 1 ha plot, distinguishing between emergent and understorey trees using the above routine should provide a much more realist representation of the composition of the canopy as viewed from atop.

Of the 45,214 stems recorded across the 36 1 ha plots, 16,253 were classified as emergent (35.9% of the total), with the vast majority of understorey trees belonging to small diameter classes (*D* < 20 cm; Fig. S1a). As expected, we found that by excluding understorey trees the estimated tree species richness of each plot was 26.3% lower on average (Fig. S1b). However, we also found that the species richness of emergent trees was strongly correlated to that of all trees within a plot (Pearson correlation coefficient (*ρ*) = 0.91, *P* < 0.0001; Fig. S1b). This suggests that by capturing variation in the species richness of emergent trees we are also adequately representing the community as a whole.

Note that in contrast to species richness, plot-level mean wood density values were estimated using data for all stems within a plot (rather than only those of emergent trees). This was done to ensure consistency with the estimates of aboveground carbon density (of which wood density is component) calculated for each plot. However, we note that because plot-level wood density estimates explicitly account for the relative basal area of each species within a plot (i.e., they give more weight to species that dominate the canopy), estimates of plot-level wood density calculated using data for all stems and those obtained using only trees classified as emergent were almost identical (*ρ* > 0.99).


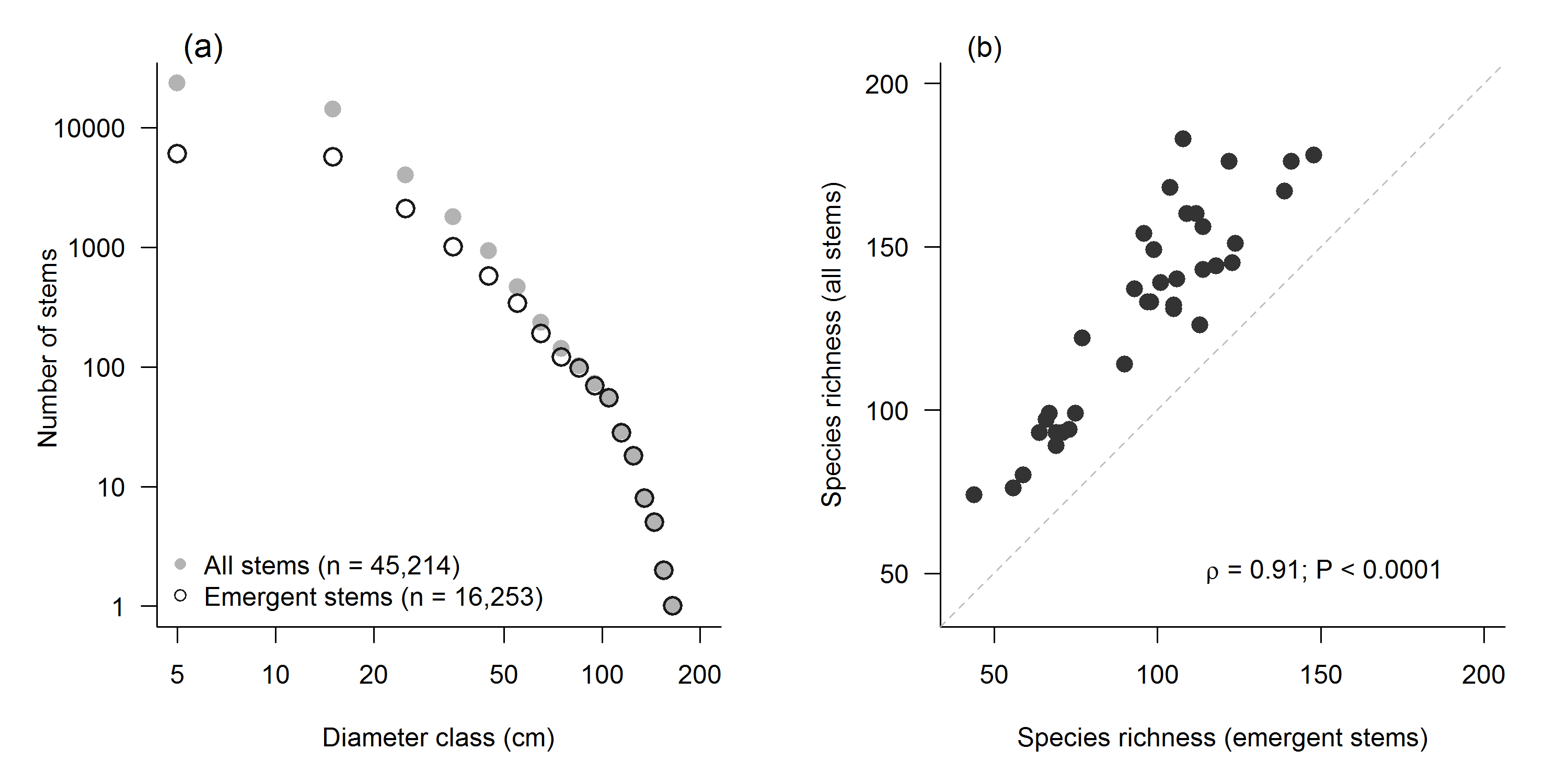
**Fig. S1**: Differences in (**a**) stem diameter distributions and (**b**) species richness when accounting for all stems and only those classified as emergent across the 36 1 ha plots. In panel (**b**) the dashed line corresponds to a 1:1 relationship and the Pearson correlation coefficient (*ρ*) between the two estimates of species richness is shown in the bottom right-hand corner.

## Appendix S2 | Selecting number of components for partial least squares regression models

To determine the optimal number of components for the partial least squares regression (PLSR) models of species richness and wood density, we calculated the root mean squared error of prediction (RMSEP) for models with varying number of components (0 to 10). RMSEP is derived from the predictive error sum of squares (PRESS) statistic, and is calculated by summing the square of the prediction errors of the model obtained for an independent validation dataset (based on leave-one-out cross validation in our case).

The PRESS statistic is calculated as:

| $\mathrm{PRESS}=\sum_{i=1}^{n} \left( y_{i}-\hat{y}_{i} \right)^{2}$ |  | (eqn S2) |
| --- | --- | --- |

where $y_{i}$ is the observed value of the response and $\hat{y}_{i}$ is the predicted value obtained from the PLSR model. Based on this, RMSEP is calculated as:

| $\mathrm{RMSEP}=\sqrt{\frac{PRESS}{n}}$ |  | (eqn S3) |
| --- | --- | --- |

where *n* is the number of observations.

When comparing PLSR models of species richness and wood density fit with varying numbers of components, we found that in the case of species richness RMSEP was lowest when using 6 components, while for wood density the optimal number of components was 7 (Fig. S2).


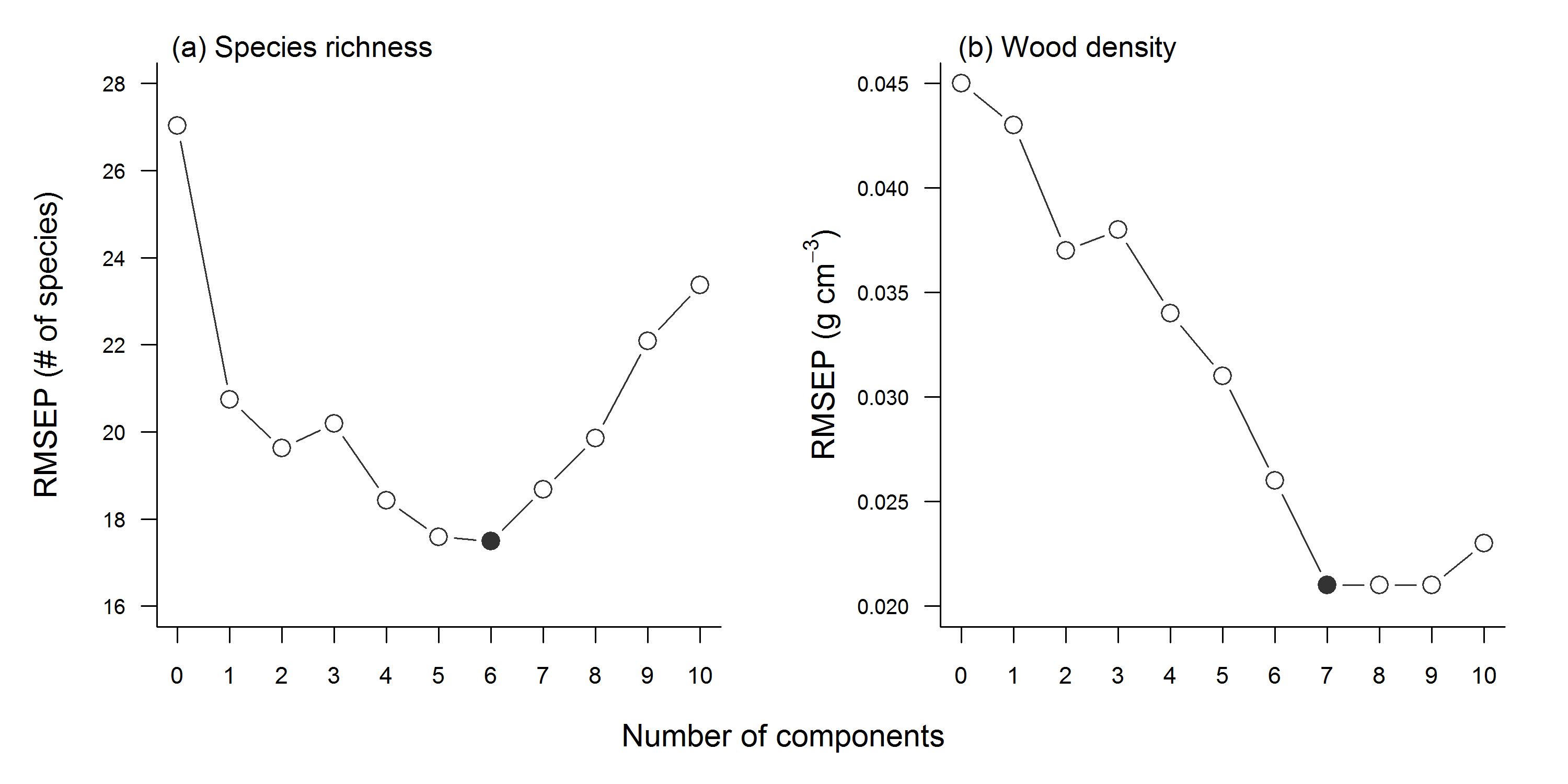


**Fig. S2**: Root mean squared error of prediction (RMSEP) values for partial least squares regression models of (**a**) species richness and (**b**) wood density fit with different numbers of components (0 to 10). Filled circle indicate the number of components that minimised RMSEP.

## Appendix S3 | Estimating ACD and basal area from ALS-derived metrics

For Sepilok, the best fitting model of aboveground carbon density (ACD; Mg C ha^-1^) using plot-measured basal area (BA; m^2^ ha^-1^) and community-mean wood density (WD; g cm^-3^) values along with ALS-derived measurements of top-of-canopy height (TCH; m) as inputs was:

| $ACD= 2.541\times{BA}^{0.974}\times{TCH}^{0.332}\times{WD}^{0.312}$ |  | (eqn S4) |
| --- | --- | --- |

The model explained 91% of the variation in plot-level ACD values and had an RMSE of 11.9 Mg C ha^-1^.

The best fitting model of plot-level BA as a function of ALS-derived gap fraction at 20 m aboveground (GF_20_) was:

| $BA=24.567\times{{GF}_{20}}^{-0.233}$ |  | (eqn S5) |
| --- | --- | --- |

The model explained 62% of the variation in plot-level BA values and had an RMSE of 3.7 m^2^ ha^-1^.

## Appendix S4 | Landscape-scale maps of topographic, structural and compositional variables


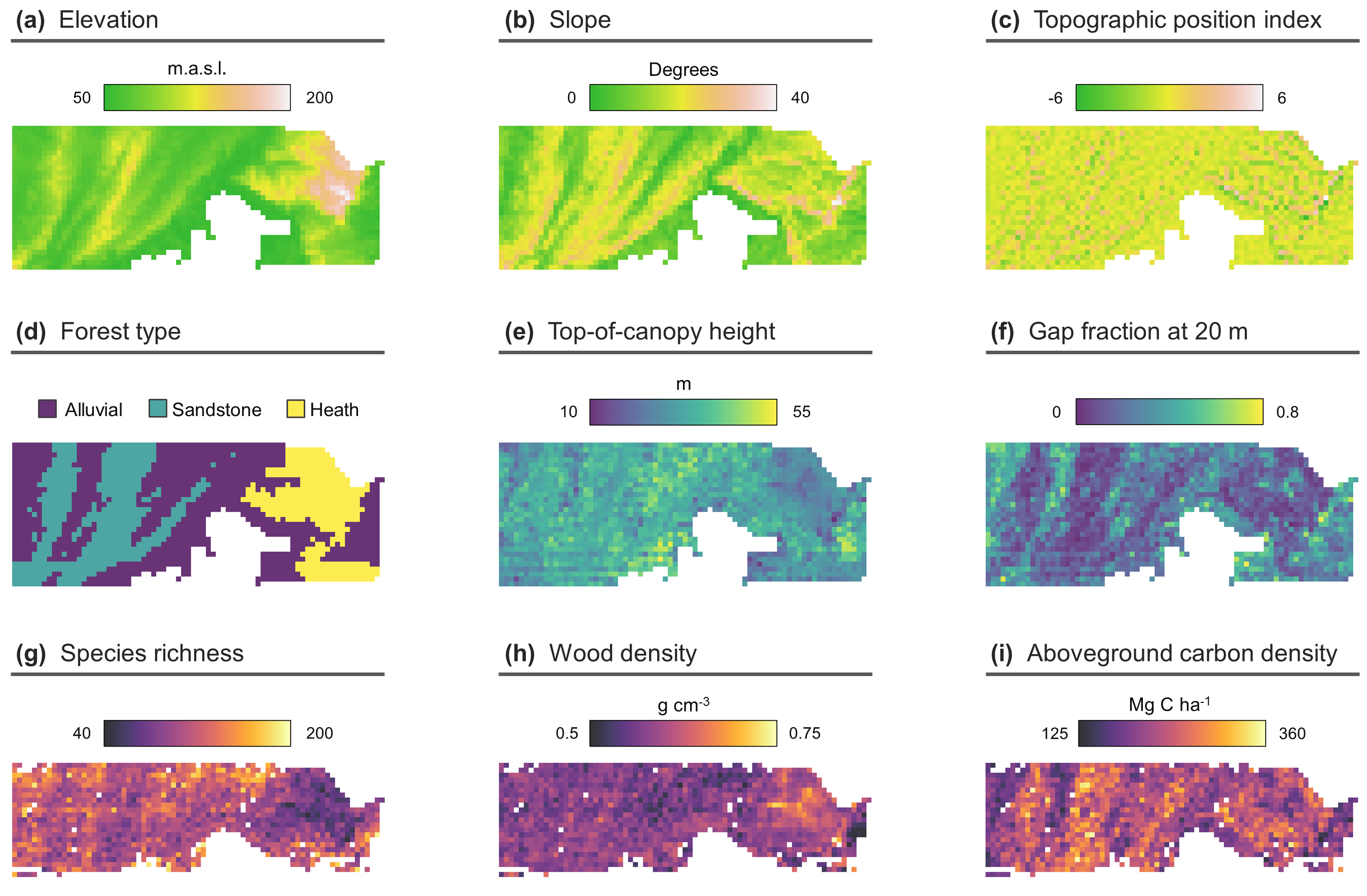


**Fig. S3**: 1-ha scale maps of topographic variables, forest structural and compositional attributes and aboveground carbon density (ACD) across Sepilok Forest Reserve. Note that for species richness, wood density and ACD, 1-ha cells with missing values correspond to areas where hyperspectral imagery was either not available or of insufficient quality to calibrate the models described in the main text.

## Appendix S5 | Summary output of best-fitting multiple regression model of ACD

**Table S1**: Summary output of best-fitting multiple regression model relating variation in aboveground carbon density (ACD) to elevation, slope, topographic position index (TPI) and species richness. Note that while the quadratic term for slope was not statistically significant in the model, model selection based on AIC supported the inclusion of the term in the final model.

| **Predictor** | **Coefficient estimate** | **Standard error** | ***t*-value** | ***P*-value** |
| --- | --- | --- | --- | --- |
| (Intercept) | 26.167 | 20.216 | 1.294 | 0.196 |
| Elevation | 1.843 | 0.269 | 6.840 | <0.0001 |
| Elevation^2^ | -0.007 | 0.001 | -6.083 | <0.0001 |
| Slope | 3.933 | 0.611 | 6.433 | <0.0001 |
| Slope^2^ | -0.094 | 0.017 | -5.598 | <0.0001 |
| TPI (absolute value) | -2.592 | 1.439 | -1.802 | 0.072 |
| Species richness | 1.129 | 0.265 | 4.266 | <0.0001 |
| Species richness^2^ | -0.005 | 0.001 | -4.653 | <0.0001 |

**Table S2**: Variation in the model coefficient estimates reported in Table S1 among forest types. Model coefficients were estimated for each forest type by fitting a mixed effects model in which forest type was treated as a random effect and all model coefficients were allowed to vary among forest types (i.e., a mixed effects model with random intercepts and slopes). Note that while doing so allowed us to estimate different parameter estimates for each forest type, we found no statistical support for doing so (ΔAIC = 5.6 when comparing the forest type-specific model to one fit to all data together). We therefore caution against interpreting the parameter estimates reported below as reflecting strong differences among forest types.

|  | **Alluvial** | **Sandstone** | **Heath** |
| --- | --- | --- | --- |
| Elevation | 2.001 | 1.029 | 0.075 |
| Elevation^2^ | -0.013 | -0.001 | 0.000 |
| Slope | 3.251 | 8.239 | 5.505 |
| Slope^2^ | -0.077 | -0.218 | -0.146 |
| TPI (absolute value) | -2.149 | -0.196 | 0.617 |
| Species richness | 0.565 | 0.289 | 1.681 |
| Species richness^2^ | -0.003 | -0.003 | -0.008 |

## Appendix S6 | Forest type-specific structural equation models


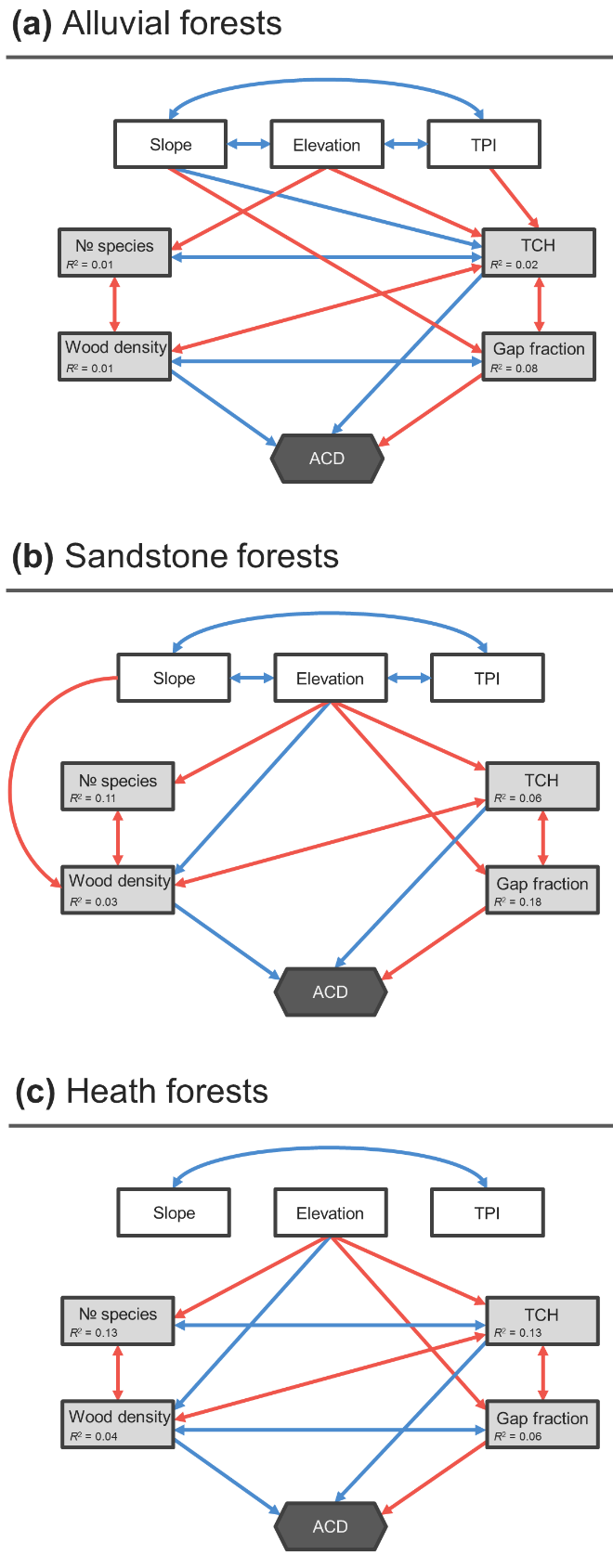


**Fig. S4**: Structural equation models (SEMs) relating variation in forest structural and compositional attributes to topography for each forest type. Model structure follows that described in the main text, but SEMs were fit separately to data for each forest type (the spatial distribution of which is shown in Fig. S3d). As in Fig. 4 of the main text, blue arrows denote positive relationships, while red arrows correspond to negative ones. R^2^ values are reported for each endogenous variable. Only statistically significant (*P* ≤ 0.05) model pathways are shown.

## References

Coomes, D.A., Dalponte, M., Jucker, T., Asner, G.P., Banin, L.F., Burslem, D.F.R.P., Lewis, S.L., Nilus, R., Phillips, O., Phuag, M.-H. & Qiee, L. (2017) Area-based vs tree-centric approaches to mapping forest carbon in Southeast Asian forests with airborne laser scanning data. *Remote Sensing of Environment*, **194**, 77–88.
